# Supplementary material for: Rapid and Widespread Distribution of Intranasal Small Extracellular Vesicles Derived from Mesenchymal Stem Cells throughout the Brain Potentially via the Perivascular Pathway
Source: Pharmaceutics. 2023 Nov 3;15(11):2578. doi: 10.3390/pharmaceutics15112578 (PMC10675165; doi:10.3390/pharmaceutics15112578)
Supplement: Supplementary file 1 [file pharmaceutics-15-02578-s001.zip › pharmaceutics-2647606-supplementary.pdf]

Rapid and Widespread Distribution of Intranasal Small Extracellular Vesicles Derived from Mesenchymal Stem Cells throughout the Brain Potentially via the Perivascular Pathway

Weiwei Shen †, Tongyao You †, Wenqing Xu, Yanan Xie, Yingzhe Wang and Mei Cui *

Department of Neurology, Huashan Hospital, Fudan University, Shanghai 200031, China; 20211220030@fudan.edu.cn (W.S.); tyyou15@fudan.edu.cn (T.Y.); 21211220027@m.fudan.edu.cn (W.X.); ynxie16@fudan.edu.cn (Y.X.); jnzhe@163.com (Y.W.)

* Correspondence: cuimei@fudan.edu.cn

† These authors contributed equally to this work.

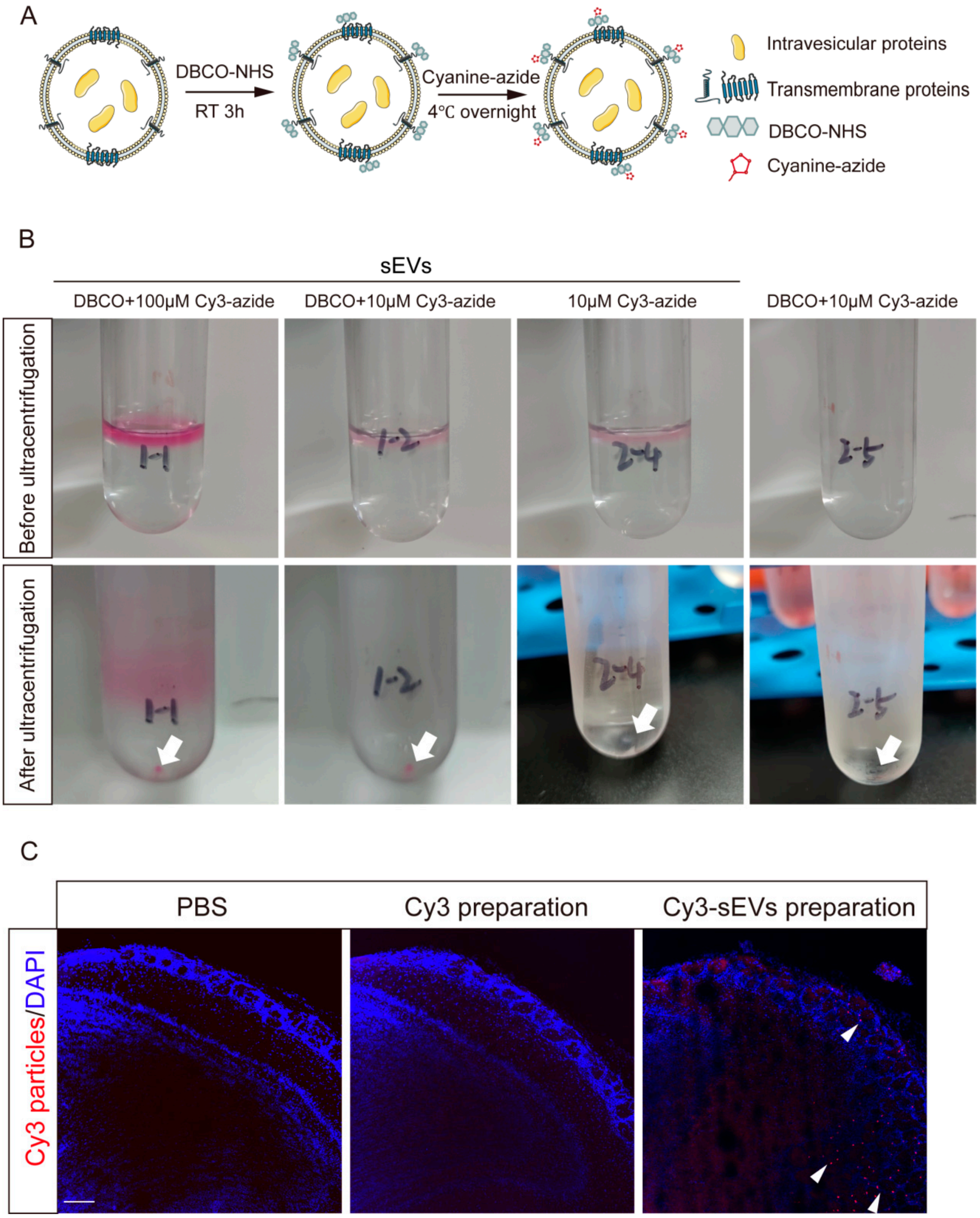

Figure S1: Labeling MSC-sEVs by click chemistry. (A) The schematic diagram of labeling methods. (B) Representative images showing the separation of sEVs from the extra dye. The white arrows indicated the location of the precipitate after ultracentrifugation. Based on an equivalent amount of sEVs, surplus dye persisted at the interface between sucrose and PBS after ultracentrifugation, while the labeled sEVs formed a precipitate on the side wall. The cyanines were unable to bind to the vesicle membrane in the absence of DBCO, a key mediator in the copper-free click chemistry reaction. When the same amount of Cy3 in the absence of sEVs was ultracentrifugated, they failed to form a precipitate as what occurred in the presence of sEVs. (C)

Representative images showing the distribution of Cy3 particles (red dots) in the presence or absence of sEVs after intranasal administration (scale bar 100 μm). Compared with the Cy3-sEVs preparation group, both the PBS group and the Cy3 preparation group (Cy3 being ultracentrifugated in the absence of exosomes) exhibited minimal fluorescence of exosomes in olfactory bulbs after intranasal administration. The white arrowheads indicated Cy3-MSC-sEVs.

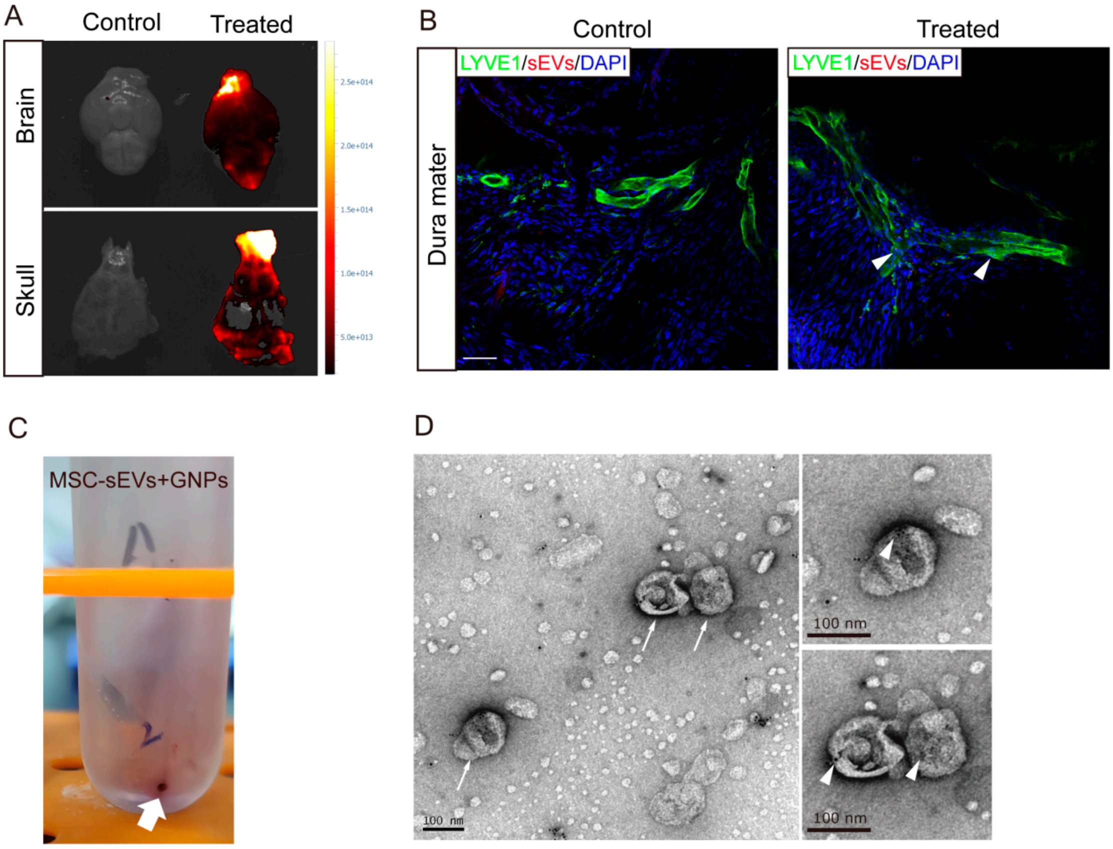

Figure S2: Distribution of MSC-sEVs and characterization of GNP-MSC-sEVs. (A) Representative images of Cy7-MSC-sEVs fluorescence in the brain (from ventral view) and the skull of the treated group at 2 h after intranasal delivery. (B) Representative images showing the presence of Cy3-MSC-sEVs (red/yellow dots) in the dura mater of the treated group (scale bar 100 μm). The white arrowheads indicate the presence of Cy3-MSC-sEVs within the cytoplasm of the lymphatic endothelium. (C) Pellet of GNP-MSC-sEVs (white arrow) obtained after ultracentrifugation. (D) Representative images of TEM showing MSC-sEVs labeled with GNPs. The white arrows indicated MSC-sEVs labeled with GNPs (left), and the white arrowheads showed magnified views of loaded GNPs (right) (scale bar 100 nm).
